# Bochdalek hernia with liver herniation

**DOI:** 10.1002/ccr3.5534

**Published:** 2022-03-03

**Authors:** Mao Kanatani, Hirokazu Taniguchi, Kimiho Kusabiraki, Yasuaki Masaki, Takeshi Tsuda, Hitoshi Abo

**Affiliations:** ^1^ The Department of Diagnostic Radiology Toyama Prefectural Central Hospital Toyama Japan; ^2^ The Department of Respiratory Medicine Toyama Prefectural Central Hospital Toyama Japan

**Keywords:** Bochdalek hernia, liver herniation, three‐dimensional image

## Abstract

Bochdalek hernia with liver herniation is rare and mimics a pulmonary mass. This case was hospitalized for masses found in the lower field of the right lung on a chest radiograph. The patient was diagnosed with Bochdalek hernia with liver herniation with three‐dimensional images created from thoracoabdominal‐enhanced computed tomography.

## Graphical abstract

This manuscript provides an important three‐dimensional (3D) image of a rare case of Bochdalek hernia with liver herniation. 3D images are useful for the diagnosis of this condition.

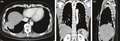

## 1 CASE PRESENTATION

**FIGURE 1:**
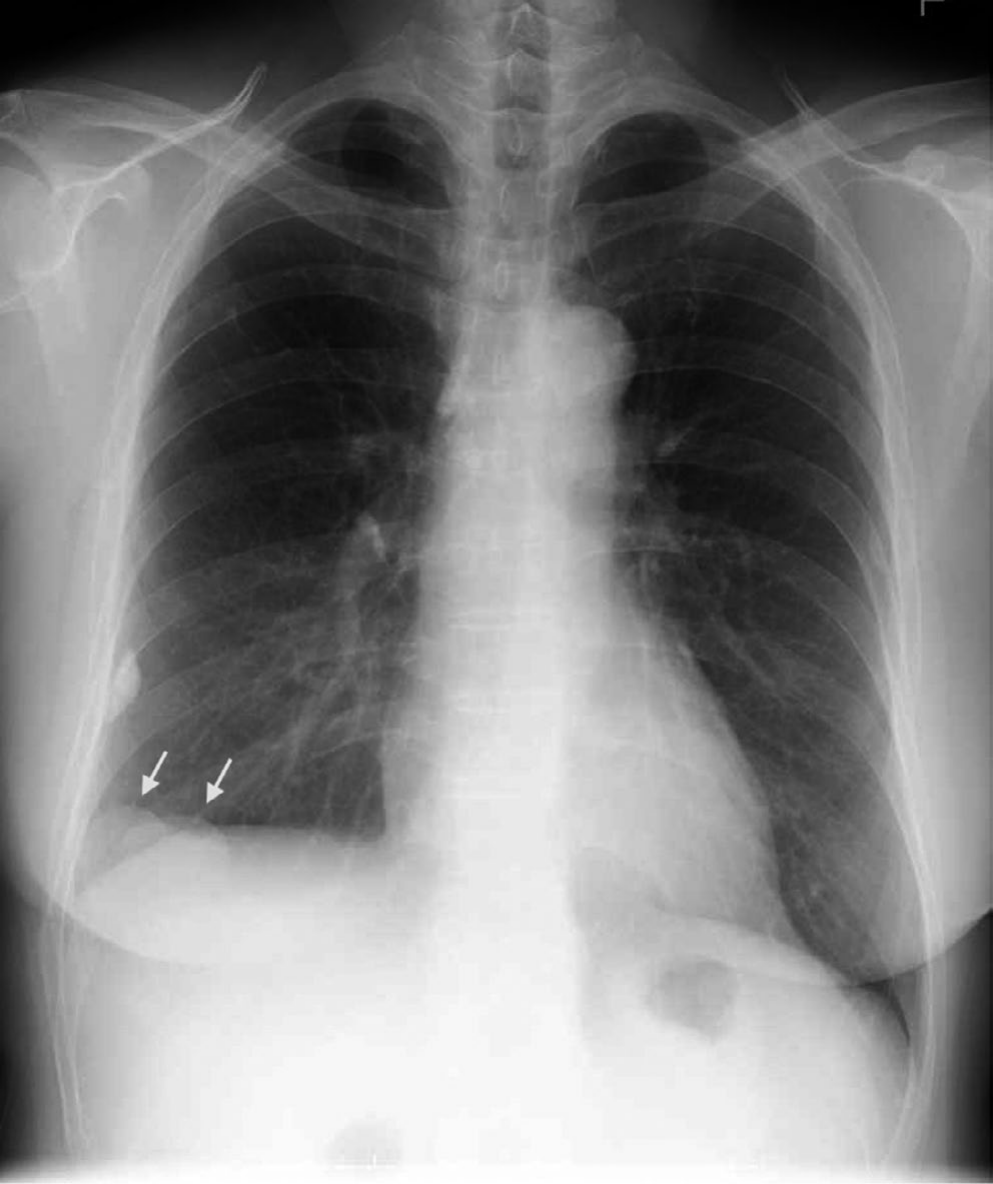
Chest radiography showed two masses in the lower field and a calcified nodule in the middle field of the right lung. Arrows indicate liver herniations

**FIGURE 2:**
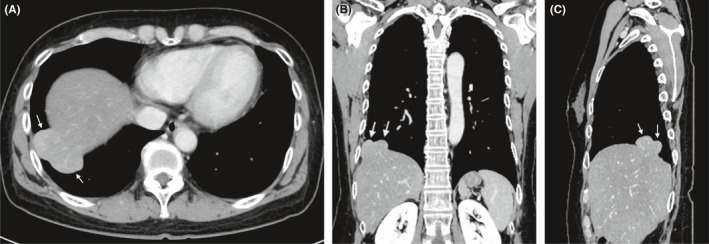
Thoracoabdominal‐enhanced computed tomography revealed smooth‐bordered masses located next to the liver. (A) Axial view, (B) coronal view, (C) sagittal view. Arrows indicate liver herniations

**FIGURE 3:**
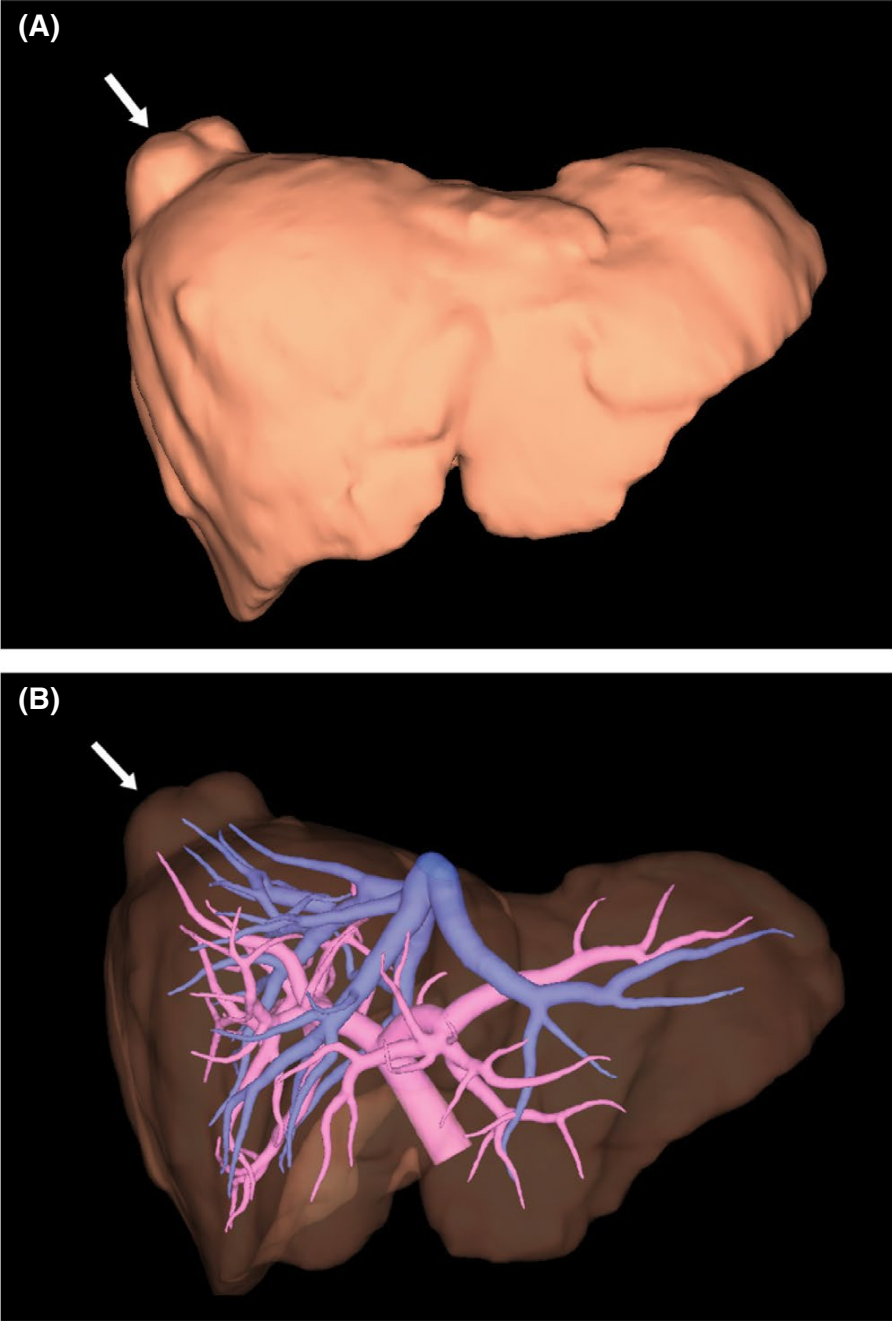
Three‐dimensional images revealed that the masses appeared to be contiguous with the liver and that the blood vessels of the masses were connected with those of the liver. (A) Overview image, (B) liver and blood vessels. Arrows indicate liver herniations

A 60‐year‐old Japanese female was hospitalized for surgery for rectal cancer, and abnormalities were found on a routine chest X‐ray before surgery. The masses were found in the lower field of the right lung on a chest radiograph (Figure 1). She had no history of respiratory problems or associated symptoms. Thoracoabdominal‐enhanced computed tomography (CT) revealed some smooth‐bordered masses located next to the liver (Figure 2). Three‐dimensional (3D) images obtained using 3D image visualization technologies revealed that the masses appeared to be contiguous with the liver and that the blood vessels of the masses were connected with those of the liver (Figure 3). As hepatic herniation may be a deformity of the liver, the blood vessels of the liver also supply blood circulation to the hernia. According to these findings, the patient was diagnosed with Bochdalek hernia with liver herniation. Our evaluation revealed no other visceral anomalies. We did not administer treatment for the hernia.

Bochdalek hernia in an adult is typically observed on the left side of the diaphragm. Bochdalek hernia with liver herniation is rare and mimics a pulmonary mass.^1^ In recent years, surgeons have used 3D image visualization technologies to improve the safety of actual surgeries. 3D images are useful for the diagnosis of Bochdalek hernia with liver herniation.^2^

## CONFLICT OF INTEREST

None declared.

## AUTHOR CONTRIBUTIONS

MK: involved in drafting of manuscript and created the computed tomography images. HT: involved in drafting of manuscript and provided patient care. KK and HA: created the computed tomography images. YM and TT: provided patient care.

## ETHICAL APPROVAL

This report was approved by the ethics committee at the Toyama Prefectural Central Hospital, and informed consent was obtained.

## CONSENT

Written informed consent was obtained from the patient to publish this report in accordance with the journal's patient consent policy.

## Data Availability

Data sharing not applicable to this article as no datasets were generated or analysed during the current study.
